# Alcohol Use Disorder Diagnoses and HIV Preexposure Prophylaxis Adherence and Continuation: a Retrospective Cohort Study

**DOI:** 10.1007/s11606-025-10114-7

**Published:** 2026-01-27

**Authors:** Anton L. V. Avanceña, Godwin Okoye, Rishit Yokananth, Aliza Norwood, Phillip W. Schnarrs, Jamie C. Barner

**Affiliations:** 1 Health Outcomes Division, College of Pharmacy, The University of Texas at Austin, 2409 University Ave, PHR 2.112, Austin, TX 78712 USA; 2 Department of Internal Medicine, Dell Medical School, The University of Texas at Austin, Austin, TX USA; 3 Institute for Community Health Research, College of Natural Sciences, The University of Texas at Austin, Austin, TX USA; 4 Department of Neuroscience, College of Natural Sciences, The University of Texas at Austin, Austin, TX USA; 5 Department of Population Health, Dell Medical School, The University of Texas at Austin, Austin, TX USA; 6 Department of Behavioral and Community Health Sciences, School of Public Health, University of Pittsburgh, Pittsburgh, PA USA

## Abstract

**Importance:**

Alcohol use disorder (AUD) has been associated with reduced adherence to and discontinuation of HIV preexposure prophylaxis (PrEP), potentially compromising its effectiveness.

**Objective:**

This study examines the relationship between AUD and PrEP adherence and continuation.

**Design:**

Retrospective cohort study using MarketScan Commercial Claims data.

**Participants:**

We included individuals aged 16–64 who initiated PrEP between January 1, 2014, and December 31, 2021, and had continuous insurance coverage.

**Main Measures:**

AUD diagnosis was identified within six months before PrEP initiation. Adherence was measured using the proportion of days covered (PDC) over 180 days and categorized by clinically relevant thresholds (≥ 85%, ≥ 80%, ≥ 57%). Continuation was assessed based on uninterrupted PrEP supply without a ≥ 30-day gap. Statistical analyses included propensity-score matching and regression modeling.

**Key Results:**

Among 43,913 eligible individuals, 1,245 (2.84%) had an AUD diagnosis prior to PrEP initiation. In a matched sample of 1,153 individuals, those with AUD had lower mean PDC (59.54% [34.14] vs. 65.85% [33.18]; *p* < 0.001) and fewer mean days of continuous PrEP use (107 [67.20] days vs. 119.90 [65.21] days; *p* < 0.001) compared to the Without AUD group. Regression analyses showed individuals with AUD had 6.31% lower mean PDC (95% CI: -9.05% to -3.57%; *p* < 0.001) and 12.93 fewer days of PrEP continuity (95% CI: -18.34 to -7.52 days; *p* < 0.001) compared to the Without AUD group. Findings may not be generalizable beyond commercially insured individuals, and we may have not captured all factors that influence PrEP adherence.

**Conclusion:**

Individuals with AUD before PrEP initiation exhibited lower adherence and continuation compared to those without AUD. Targeted interventions may be required to enhance PrEP adherence and continuity in this population.

**Supplementary Information:**

The online version contains supplementary material available at https://doi.org/10.1007/s11606-025-10114-7.

## INTRODUCTION

New diagnoses of HIV in the US have remained stable between 2017 (38,433) and 2023 (38,850) and remain far higher than the national target of 3,000 new cases by 2030 [1–3]. These trends highlight the importance of expanding access to primary prevention strategies, such as HIV pre-exposure prophylaxis (PrEP). PrEP—HIV medications that have been approved for prevention—have been recommended by the Centers for Disease Control and Prevention (CDC) and the US Preventive Services Task Force for adults and adolescents who face substantial risk of HIV [4, 5]. The effectiveness of PrEP is contingent on adherence (taking medication as advised or indicated) and continuation or persistence (uninterrupted medication use or supply). Consistent use of PrEP can reduce the risk of HIV from sex by 99% and from injection drug use by 74% [6, 7]. However, nonadherence can reduce or eliminate PrEP’s protective effect. A CDC analysis of trial data found that when oral PrEP adherence fell to approximately 40% or lower, its association with HIV risk reduction was no longer statistically significant (RR 0.93, 95% CI: 0.72–1.20) [6].

Alcohol use, particularly unhealthy consumption, is one of several factors that affect PrEP adherence and continuation—two steps in the PrEP care continuum [8]. For example, men who have sex with men (MSM) in Toronto who engaged in unhealthy alcohol use, defined as having an Alcohol Use Disorders Identification Test (AUDIT) score of ≥ 16, had nearly 7 times higher odds of nonadherence (95% CI: 1.49–30.33; p = 0.013) compared to low-risk drinkers [9]. In Kenya, a study found that alcohol use disorder (AUD), a category of unhealthy alcohol use [10], was associated with discontinuation among MSM and transgender women who initiated PrEP [11]. Other studies, however, have found that alcohol use does not affect PrEP use, suggesting that individuals who use alcohol can use PrEP effectively [12–16].

To build on this literature, we sought to assess the effect of AUD diagnoses on PrEP adherence and continuation using large, multi-year data from a real-world (i.e., non-trial) patient population. While most prior studies on this topic have focused on self-reported alcohol use or AUD symptoms (measured via AUDIT), we focused on clinical AUD diagnoses, which represent an important and necessary step in treating AUD [17]. Findings from this study can inform PrEP care guidelines and identify the need for interventions that address AUD and PrEP use concurrently.

## METHODS

### Data Source

This retrospective cohort study followed the STROBE reporting guideline [18]. We used Merative MarketScan® data, which contains a convenience sample of > 25 million individuals per year with employer-sponsored commercial health insurance and their dependents. The data includes demographic information, diagnoses in *International Classification of Diseases, Ninth and Tenth Revision* (ICD-9 and ICD-10) codes, and records for inpatient and outpatient services and outpatient prescriptions. Because MarketScan data are de-identified, this study was exempted from full review by The University of Texas Institutional Review Board (STUDY00006393).

### Study Population

We included individuals aged 16–64 years at the time of PrEP initiation from January 1, 2014, to December 31, 2021. PrEP initiation was determined through an algorithm that has been adopted in previous studies involving real-world data [19]. First, we identified pharmacy claims for Food and Drug Administration (FDA)-approved medications for PrEP: 2 oral combination medications—tenofovir disoproxil fumarate/emtricitabine (TDF/FTC) and tenofovir alafenamide/emtricitabine—and a long-acting injectable (cabotegravir). These medications were identified using National Drug Codes for the formulations used in PrEP (eTable 1 in Supplement 1). Second, we excluded patients diagnosed with HIV or hepatitis B, which are other indications for TDF/FTC (eTable 2 in Supplement 1). Consistent with other studies that use real-world-data [20, 21], we further limited the sample to individuals that had 6 months of continuous insurance coverage before and after the date of their PrEP initiation (i.e., the index date).

We classified our sample based on their AUD diagnosis history, which we assessed using ICD-9 and ICD-10 codes in inpatient and outpatient service claims, as previous claims-based studies have also done (eTable 3 in Supplement 1) [22–25]. Individuals who had an AUD diagnosis in the 6 months before PrEP initiation were included in the “Pre-initiation AUD” group. Individuals without AUD diagnoses in the baseline and follow-up periods were included in the “Without AUD” group. Due to the underdiagnosis of AUD and the potential for misclassification of AUD status in claims data [26, 27], we also identified individuals who received an AUD diagnosis in the 6 months after PrEP initiation (“Post-initiation AUD” group) as a supplemental analysis. Previous research has found that individuals diagnosed with AUD after PrEP initiation have similar characteristics as those who were diagnosed before initiation [28], suggesting that these individuals may have been experiencing AUD symptoms before starting PrEP.

## MEASURES

### Outcomes

Our primary outcomes were PrEP adherence and continuation. Adherence refers to the degree to which individuals follow the prescribed dosing regimen for their PrEP medication over time [29]. In this study, we measured adherence using proportion of days covered (PDC), calculated by dividing the number of days an individual has PrEP medications by the follow-up period (180 days) using pharmacy claims. We also created 3 binary adherence outcomes using clinically relevant PDC thresholds. The first was PDC ≥ 85%, representing an average of 6 doses per week for oral PrEP and about 4 doses of long-acting PrEP over 6 months [29]. An 80% PDC threshold is the most widely used cutoff in medication adherence studies and is recommended by the Pharmacy Quality Alliance [30]. Finally, we identified individuals who had a PDC ≥ 57%, which represents having 4 doses of oral PrEP per week (or 2 doses of long-acting PrEP)—the minimum threshold known to provide effective protection to cisgender males, who are the most common PrEP users [31]. This lower PDC threshold also captures individuals who follow “on-demand” or “event-driven” PrEP dosing, which involves taking 2 PrEP pills before a sexual encounter and 1 daily PrEP pill for the following 2 days [29].

We also assessed continuation or persistence, which refers to the consistency with which individuals remained on PrEP without a significant gap over a specified follow-up period [32]. We measured continuation by calculating the days individuals had PrEP from their index date to 180 days after initiation. Individuals were classified as having discontinued PrEP if they had a ≥ 30-day gap in sufficient PrEP coverage. For individuals who received long-acting PrEP, this represented missing 1 injectable dose following the first 2 loading doses.

### Covariates

Sociodemographic characteristics, including age, sex assigned at birth, insurance plan, employment status, and geographic region, were measured on the index date. We also captured several clinical characteristics, including diagnoses of depression, anxiety, bipolar disorder, and substance use disorders (SUDs) related to the use of opioids, nicotine, cannabis, stimulants, sedatives, and other substances that have been associated with PrEP nonadherence in prior studies using ICD-9 and ICD-10 codes (eTables 4–5 in Supplement 1) [9, 33]. We also assessed diagnoses of STIs and use of STI screening and psychotherapy services (eTables 6–7 in Supplement 1).

To minimize the effect of measured confounders, we generated a matched cohort of individuals who use PrEP with and without AUD diagnoses using propensity scores. We first calculated each individual’s propensity score using multivariable logistic regression, including all sociodemographic and clinical characteristics as predictors of the probability of receiving an AUD diagnosis. Then, we matched individuals in the Pre-initiation AUD diagnosis and Without AUD diagnosis groups (the exposed and control groups) with a 1:1 ratio based on their propensity scores. We used greedy nearest neighbor matching without replacement and a 0.01 caliper width to ensure close matches. We also matched individuals in the Post-initiation AUD group with those in the Without AUD group. To evaluate the quality of the matching process, we assessed standardized mean differences (SMDs) of the covariates and used a threshold of SMD < 0.1 for covariate balance.

### Statistical Analysis

We used descriptive statistics to summarize baseline characteristics separately for individuals who use PrEP with and without AUD diagnosis. Statistical differences between two groups were evaluated using Student’s t-test for continuous variables, Pearson’s chi-square test for binary variables, and Mantel–Haenszel chi-square test for nominal variables.

We also used descriptive statistics to summarize our outcomes. For adherence, we calculated the mean (standard deviation [SD]) PDC and reported the proportion of individuals that were considered adherent to PrEP based on 3 thresholds (85%, 80%, and 57%). For continuation, we calculated the mean (SD) days individuals had continuous or uninterrupted PrEP use.

To estimate the effect of AUD diagnosis on adherence and continuation, we conducted multivariable regression analyses using the propensity score-matched cohort of individuals who use PrEP. We used a logistic regression model to compare our binary adherence outcome among individuals with and without AUD diagnoses. We also used linear regression model to assess the association between AUD diagnoses and PDC and continuation among individuals who use PrEP.

## RESULTS

### Baseline Characteristics

Among 132,737 individuals who had claims for at least one FDA-approved PrEP, 43,913 met the full inclusion criteria. The most common type of PrEP received by individuals in the Pre-initiation AUD and No AUD group was TDF/FTC (87.71% and 86.36%, respectively), followed by TAF/FTC (12.29% and 13.64%, respectively; eFigure 1 in Supplement 1). We did not identify any individuals who used long-acting PrEP (cabotegravir).

1,245 (2.84%) individuals were included in the Pre-initiation AUD group and 37,639 (85.71%) individuals in the Without AUD group, who differed significantly in their baseline characteristics (Table 1). Compared to the Without AUD group, individuals in the Pre-initiation AUD group were younger, had a lower proportion of males assigned at birth 83.3% vs 90.3%, lower proportion of employed individuals, and a higher proportion with prior diagnoses of mental illness and SUDs. The matching process identified 1,153 matched pairs of individuals in the Pre-initiation AUD and Without AUD groups (Table 1; eFigure 2 in Supplement 1). Individuals in the Pre-initiation AUD group who were not matched (*n* = 92 or 7.4%) were younger, more likely to be male, and had more mental health comorbidities than individuals who were matched (eTable 9 in Supplement 1).

**Table 1:** Baseline demographic and clinical characteristics of unmatched and matched individuals who use PrEP by AUD diagnosis status (2014–2021)

Characteristics	Unmatched				Matched		
	Pre-initiation AUD group (*N* = 1,245)	Without AUD group (*N* = 37,639)	SMD^a^	Pre-initiation AUD group (*N* = 1,153)		Without AUD group (*N* = 1,153)	SMD^a^
Age in years, mean (SD)	34.9 (11.4)	35.9 (10.8)	0.087	35.4 (11.2)		35.3 (11.5)	0.005
Sex assigned at birth, n (%)							
Male	1,037 (83.3)	33,990 (90.3)	0.087	981 (85.1)		975 (84.6)	0.002
Female	208 (16.7)	3,649 (9.7)		172 (14.9)		178 (15.4)	
Insurance plan, n (%)			0.063				0.035
HMO	176 (14.1)	5,015 (13.3)		142 (12.3)		166 (14.4)	
POS	231 (18.6)	7,539 (20.0)		230 (19.9)		217 (18.8)	
PPO	584 (46,9)	16,987 (45.1)		543 (47.1)		535 (46.4)	
Others	254 (20.4)	8,098 (21.5)		238 (20.6)		235 (20.4)	
Employed, n (%)	986 (79.2)	31,409 (83.5)	0.109	907 (78.7)		914 (79.3)	0.016
Region^b^, n (%)			N/A				N/A
Northeast	348 (27.9)	9,531 (25.3)		332 (28.9)		315 (27.5)	
Midwest	175 (14.0)	4,833 (12.8)		149 (13.0)		160 (13.9)	
South	428 (34.4)	13,897 (36.9)		401 (34.9)		404 (35.2)	
West	288 (23.1)	9,253 (24.6)		266 (23.2)		268 (23.4)	
Unknown	6	125		5		6	
Depression, n (%)	389 (31.2)	1996 (5.3)	0.713	333 (28.9)		316 (27.4)	0.004
Anxiety, n (%)	333 (26.8)	2,488 (6.6)	0.561	293 (25.4)		280 (24.3)	0.031
Bipolar, n (%)	162 (13.0)	590 (1.6)	0.451	109 (9.5)		118 (10.2)	0.031
PTSD, n (%)	94 (7.6%)	738 (2.0%)	0.265				0.021
Schizophrenia, n(%)	10 (0.8)	28 (0.1)	0.110	5 (0.4)		9 (0.8)	0.053
Substance use disorders			0.888				0.023
Opioids, n (%)	65 (5.2)	90 (0.2)		48 (4.1)		18 (1.5)	
Nicotine, n (%)	164 (13.8)	476 (1.3)		125 (10.9)		177 (15.3)	
Cannabis, n (%)	121 (9.7)	116 (0.3)		86 (7.4)		68 (5.9)	
Stimulants, n (%)	100 (8.0)	103 (0.3)		82 (7.1)		49 (4.2)	
Sedatives, n (%)	64 (5.1)	29 (0.1)		53 (4.6)		14 (1.2)	
Other, n (%)	70 (5.6)	99 (0.3)		51 (4.4)		42 (3.6)	
STI n (%)	132 (10.6)	6,700 (17.8)	0.207	115 (10.0)		125 (10.8)	0.025
STI screening n (%)	189 (15.2)	3,806 (10.1)	0.153	170 (14.7)		166 (14.4)	0.010
Psychotherapy n (%)	173 (13.9)	2,084 (5.5)	0.285	159 (13.8)		150 (13.0)	0.027

^a^An SMD < 0.1 shows adequate covariate balance

^b^Region was excluded from the propensity score model because of insufficient sample size in the unknown group

Abbreviations: *AUD*, alcohol use disorder; *HMO*, health maintenance organization; *PrEP*, preexposure prophylaxis; *POS*, point of service; *PPO*, preferred provider organization; *PTSD*, posttraumatic stress disorder; *SMD*, standardized mean difference; *STI*, sexually transmitted infection

### Adherence to PrEP

Using the matched sample, we found that the Pre-initiation AUD group had lower PrEP adherence compared to the Without AUD group (mean PDC [SD]: 59.54% [34.14] vs. 65.85% [33.18]; *p* < 0.001; Table 2). In regression analysis, we found that individuals in the Pre-initiation AUD group had 6.31% lower mean PDC (95% CI: -9.05% to -3.57%; *p* < 0.001; Table 3) compared to the Without AUD group.

**Table 2:** Comparison of adherence and continuation to HIV preexposure prophylaxis by AUD diagnosis status

	Primary analysis			Supplemental analysis		
	Pre-initiation AUD group (*N* = 1,153)	Without AUD group (*N* = 1,153)	*P*-value	Post-initiation AUD group (*N* = 4,303)	Without AUD group (*N* = 4,303)	*P*-value
Adherence						
Mean PDC (SD)%	59.54 (34.14)	65.85 (33.18)	< 0.001	63.39 (34.21)	66.89 (32.30)	< 0.001
PDC ≥ 85%, n (%)	411 (35.65)	501 (43.45)	< 0.001	1,775 (41.25)	1,883 (43.76)	0.019
PDC ≥ 80%, n (%)	473 (41.02)	577 (50.04)	< 0.001	2,048 (47.59)	2,183 (50.73)	0.004
PDC ≥ 57%, n (%)	603 (52.30)	700 (60.71)	< 0.001	2492 (57.91)	2653 (61.65)	< 0.001
Continuation, mean (SD) days	107.00 (67.20)	119.90 (65.21)	< 0.001	114.90 (67.42)	121.90 (64.07)	< 0.001

This analysis used the propensity score-matched sample of individuals with and without AUD diagnoses

Abbreviations: *AUD*, alcohol use disorder; *PDC*, proportion of days covered; *PrEP*, preexposure prophylaxis

**Table 3:** Results of regression analysis for adherence and continuation of HIV preexposure prophylaxis

Outcome	Pre-initiation AUD vs. Without AUD groups (reference)			Post-initiation AUD vs. Without AUD groups (reference)		
	OR	95% CI	*P*-value	OR	95% CI	*P*-value
Adherence						
PDC ≥ 85%	0.72	0.61 – 0.85	< 0.001	0.90	0.83 – 0.98	0.019
PDC ≥ 80%	0.69	0.59—0.82	< 0.001	0.88	0.81—0.96	0.004
PDC ≥ 57%	0.71	0.60 – 0.84	< 0.001	0.86	0.79 – 0.93	< 0.001
	**Estimate**	**95% CI**	***P*-value**	**Estimate**	**95% CI**	***P*-value**
Adherence, mean PDC (%)	-6.31	-9.05 to -3.57	< 0.001	-3.51	-4.92 to -2.10	< 0.001
Continuation, mean days	-12.93	-18.34 to -7.52	< 0.001	-6.94	-9.72 to -4.16	< 0.001

This analysis used the propensity score-matched sample of individuals with and without AUD diagnoses

Abbreviations: *AUD*, alcohol use disorder; *OR*, odds ratio; *PDC*, proportion of days covered

A lower proportion of individuals in the Pre-initiation AUD group met the 85% (35.65% vs. 43.45%; *p* < 0.001), 80% (41.02% vs. 50.04%; *p* < 0.001), and 57% (52.30% vs. 60.71%; *p* < 0.001) PDC thresholds for PrEP adherence (Table 2). Regression analyses found that the Pre-initiation AUD group had lower odds of meeting the 85% (OR 0.72; 95% CI: 0.61–0.85; *p* < 0.001), 80% (OR 0.69; 95% CI: 0.59–0.82; *p* < 0.001), or 57% (OR 0.71; 95% CI: 0.60–0.84; *p* < 0.001) PDC thresholds compared to the Without AUD group (Table 3).

### Continuation of PrEP

The Pre-initiation AUD group had fewer days of continuous PrEP medication compared to the Without AUD group (107 [67.20] days vs. 119.90 [65.21] days; *p* < 0.001; Table 2). Regression analysis estimated the Pre-initiation AUD group had 12.93 fewer days of continuous PrEP medication (95% CI: -18.34 to -7.52 days; *p* < 0.001; Table 3) compared to the Without AUD group.

### Supplemental Analysis

5,029 out of 43,913 (11.45%) individuals who use PrEP received an AUD diagnosis within 6 months after initiation and were included in the Post-initiation AUD group (eFigure 1 in Supplement 1). Individuals in the Post-initiation AUD group were younger, had a lower proportion of males assigned at birth, a lower proportion of employed individuals, and a higher proportion with prior diagnoses of mental illness and SUDs compared to the Without AUD group (eTable 10 in Supplement 1).

The matching process identified 4,303 matched pairs of individuals from the Post-initiation AUD and Without AUD groups (eFigure 3 and eTable 10 in Supplement 1). A comparison of PDCs showed that the Post-initiation AUD group had lower PrEP adherence (mean PDC [SD]: 63.39% [34.21] vs. 66.89% [32.30]; *p* < 0.001; Table 2). The proportion of individuals who met the thresholds for adherence was also higher in the Without AUD group compared to the Post-initiation AUD group (Table 2). In regression analysis, we found that individuals in the Post-initiation AUD group had 3.51% lower mean PDC (95% CI: -4.92% to -2.10%; *p* < 0.001; Table 3) compared to the Without AUD group. The Post-initiation AUD group also had fewer days with continuous PrEP claims compared to the Without AUD group (mean [SD] 114.90 [67.42] days vs. 121.90 [64.07] days; *p* < 0.001; Table 2).

Regression analysis found that the Post-initiation AUD group had lower odds of meeting the 85% (OR 0.90; 95% CI: 0.83–0.98, *p* = 0.019), 80% (OR 0.88; 95% CI: 0.81–0.96, *p* = 0.004), and 57% PDC thresholds (OR 0.86; 95% CI: 0.79–0.93, *p* < 0.001) and had 6.94 (95% CI: -9.72 to -4.16 days, *p* < 0.001) fewer days of continuous PrEP use compared to the Without AUD group (Table 3).

## DISCUSSION

This retrospective cohort study used a matched sample of individuals who initiated PrEP and found suboptimal PrEP use among those who were previously diagnosed with AUD. In our entire sample, the mean PDC was less than 70% across the AUD and non-AUD groups. However, individuals who were diagnosed with AUD before initiation had 7% lower mean PDC than their counterparts without AUD. Individuals who were diagnosed with AUD also had fewer days of continuous or “gap-free” PrEP supply. We found similar associations between AUD and PrEP adherence and continuation among those who were diagnosed with AUD after PrEP initiation.

Alcohol use has been repeatedly found to negatively affect medication adherence [34–36], likely by impairing prospective memory (i.e., “forgetfulness”) and increasing avoidance behaviors, particularly in individuals who believe alcohol interacts with their medications [37]. In HIV treatment, alcohol and substance use disorders have been found to reduce adherence to antiretroviral therapy (ART) [38–40].

Mirroring findings in ART studies, alcohol use is known to affect PrEP use. For example, in a Kaiser Permanente study (*N* = 2,995), individuals with AUD who were eligible for PrEP were 23% more likely to be linked with a PrEP provider within a year of referral but were 15% less likely to be adherent to daily PrEP use, defined as a medication possession ratio of ≥ 80% [41]. Despite similarities in study aims and approaches, our study improves on this previous analysis in several ways. First, we defined PrEP adherence using PDC, a measure that is less prone to overestimation than medication possession ratio [42]. Second, we employed propensity-score matching of individuals with and without AUD diagnoses and used data through 2021, which allowed us to include individuals who initiated TDF/FTC (approved in 2013) and TAF/FTC (approved in 2019). Finally, our use of real-world data allowed us to include a diverse cohort of individuals from all US regions who receive care from different healthcare settings.

Prior research, however, has also found null or positive associations between unhealthy alcohol use and PrEP outcomes [43, 44]. A study with a national sample of Black and Latino MSM found that higher AUDIT scores were associated with 6% lower odds of PrEP awareness but 6% higher odds of self-reported PrEP adherence, which can overestimate actual adherence [16]. In South Africa, a study with periconceptional women found that laboratory-confirmed adherence was more likely among those who reported any past-year drinking [13, 45]. These results suggest that individuals who use alcohol and may be at higher risk for HIV [46], making them ideal candidates for PrEP, can take the medication effectively and achieve optimal protection. If these findings hold, additional research should aim to understand the conditions or factors that are associated with PrEP adherence among people who use alcohol and other substances.

We also found that individuals with AUD experienced ≥ 30-day gaps in their PrEP supply about 12 days sooner than those without an AUD diagnosis. Like the literature on PrEP adherence, prior studies have yielded mixed results on the association between PrEP (dis)continuation and alcohol use [47, 48]. While gaps in PrEP supply can lower its effectiveness, other factors not related to PrEP use may impact a person’s HIV risk. For example, individuals may discontinue PrEP because of changes in their risk profiles, such as starting a monogamous relationship or adopting other prevention measures (e.g., condoms) [49, 50]. We used a 6-month follow-up to focus on the initial period of PrEP use, where significant changes in HIV risk are less likely to occur, and to identify early drop-offs that require re-engagement in PrEP care [29].

Contradicting findings on the association between alcohol use and PrEP may be influenced by several factors, such as the population (e.g., MSM vs. women), geography or location, length of follow-up, and presence of co-occurring conditions. How unhealthy alcohol use is measured or defined may also be a contributor. Studies that analyzed primary data often relied on self-reported alcohol use or responses to AUDIT, a validated screening tool, to identify individuals who have unhealthy alcohol use or AUD. While these studies may have been more sensitive to identifying individuals with AUD, self-reported alcohol use is still subject to social desirability bias [51]. By contrast, claims-based analyses like our study used AUD diagnosis codes to identify individuals with AUD [22–25]. While this approach may underestimate the burden of AUD due to coding errors and underdiagnosis, our study highlights needed improvements in the continuum of care among individuals at the intersection of HIV prevention and addiction, even if they may have fewer healthcare barriers (e.g., continuous insurance coverage).

While efforts are needed to improve PrEP use generally [52–54], our study underscores the importance of targeted efforts to address the challenges faced by people with AUD. Unfortunately, interventions developed to address unhealthy alcohol use and promote PrEP adherence and continuation have not been effective [55–58]. Most of these interventions utilized motivational interviewing approaches, web-based applications, or brief behavioral interventions. For example, Starks et al. (2024) and Karnik et al. (2023) both evaluated MI-based and brief interventions but found no statistically significant differences in alcohol use or PrEP-related outcomes between intervention and control groups. Similarly, Wray et al. (2024) tested a digital personalized feedback web app and found no improvements in PrEP adherence or alcohol outcomes compared to standard education. Common issues reported in these studies include high dropout rates, particularly in trials conducted during COVID-19, low participant engagement, and challenges in delivering interventions as intended.

An unexplored but promising avenue involves integrating alcohol AUD pharmacotherapy with PrEP adherence interventions. To date, no studies have evaluated the combined delivery of AUD pharmacotherapies (i.e., naltrexone, acamprosate, and disulfiram) alongside routine PrEP care. Newer long-acting PrEP regimens, like twice-yearly lenacapavir injections [59], may also reduce the burden on individuals to take daily pills and offer a promising alternative for individuals with AUD who face adherence issues. However, long-acting PrEP may not be a panacea. In HIV therapy, people with HIV still face adherence challenges when using long-acting ART. Studies have found that while some patients with substance use and housing instability viewed long-acting ART as a way to improve adherence, other challenges, such as missed appointments, transportation difficulties, and lifestyle mismatches associated with the resource-intensive nature of long-acting ART use, can easily undermine its effectiveness [60, 61]. Thus, long-acting formulations may not eliminate all barriers to medication adherence. Future research should examine how long-acting formulations mitigate PrEP adherence challenges among individuals with AUD.

### Limitations

Our study has several limitations. We relied on diagnosis codes to identify patients with AUD, which is subject to misclassification and underreporting [26]. Best practices for AUD diagnosis involve structured assessments and the use of validated tools like AUDIT; however, claims data such as MarketScan do not include details on the screening or diagnosis procedures followed. Some studies have sought to address this limitation by including > 1 AUD diagnoses (particularly in outpatient settings) to classify an individual as having AUD [62], but most studies have relied on the presence of 1 diagnosis of AUD in claims data [22–25]. Similarly, we measured adherence and continuation indirectly using pharmacy claims, and we could not ascertain whether patients were taking their PrEP medications. In previous research, adherence was assessed through other means, including self-reported behavior [63, 64] or pharmacological exposure measures like TFV concentrations in blood plasma, dried blood spots, and hair samples [13, 16, 65, 66]. Adherence monitoring using TFV diphosphate is often preferred because its blood concentration detects cumulative adherence over several weeks [13, 67] and is less prone to “white-coat compliance” and other forms of observer bias [65, 68]. However, while biomarker-based monitoring can confirm or validate self-reported PrEP adherence and continuation, these techniques can be costly and have little utility in real-world settings. By contrast, measuring adherence in PDC using pharmacy claims offers a low-cost alternative with high sensitivity, but low specificity, when used to assess adherence over a limited period [29].

Claims data exclude diagnoses or healthcare services received without insurance or outside traditional healthcare settings. This may be especially relevant for adolescents whose healthcare coverage is through their primary caregivers and may choose to receive PrEP and other related services (e.g., STI screening) through community access programs to avoid disclosure [69, 70]. Related to this, our algorithm for identifying individuals who take PrEP, while highly sensitive and specific [19] and routinely used for PrEP surveillance in the US [71], may have missed or misclassified individuals. MarketScan only includes individuals with employer-sponsored commercial insurance and leaves out other populations, limiting our study’s generalizability. MarketScan also lacks information on key variables such as gender, sexual orientation, race, and ethnicity, which are important determinants of both AUD and PrEP use. We also did not account for other barriers to PrEP adherence and continuation, such as medical and non-medical financial constraints, medication concerns, and other care-access barriers, which may explain the associations we found [72–74]. The absence of these variables may have introduced bias in our matching approach, which only controlled for measured confounders in estimating the association—and not the causal link—between AUD and PrEP adherence and continuation. Our matching procedure also failed to match 7.4% of our exposed individuals whose characteristics differed from those who were matched with an individual in the control group. While our strict matching criteria ensured covariate balance in our final matched sample, this approach may have contributed to lower precision (i.e., higher variance) and selection bias that future studies can address using other matching algorithms (e.g., many-to-one matching) [75, 76].

## CONCLUSION

This retrospective cohort study found that individuals who use PrEP and were diagnosed with AUD before or after PrEP initiation had lower adherence and continuation compared to those without AUD diagnoses. These findings conflict with previous research, suggesting that AUD may affect PrEP adherence and continuation in specific contexts and populations that future research should explore. Interventions, including longer-acting PrEP formulations, may be needed to help individuals with AUD to take PrEP as recommended.

## Supplementary Information

Below is the link to the electronic supplementary material.

Supplementary file1 (DOCX 238 KB)

## Data Availability

MarketScan is a licensed database of Merative and therefore not publicly available. Statistical code is available upon request to the corresponding author (AA). All inquiries should be directed to the corresponding author.
