# Supplementary material for: Alcohol Use Disorder Diagnoses and HIV Preexposure Prophylaxis Adherence and Continuation: a Retrospective Cohort Study
Source: J Gen Intern Med. 2026 Jan 27;41(14):4102–11. doi: 10.1007/s11606-025-10114-7 (PMC13728049; doi:10.1007/s11606-025-10114-7)
Supplement: Supplementary file 1 [file 11606_2025_10114_MOESM1_ESM.docx]

# SUPPLEMENTARY MATERIALS

eFigure 1. CONSORT diagram showing the cohort selection of PrEP users with and without AUD diagnoses

eFigure 2. Love plot showing standardized mean differences before and after propensity score matching for patients with AUD diagnosis before PrEP initiation vs patients without AUD

eFigure 3. Love plot showing standardized mean differences before and after propensity score matching for patients with AUD diagnosis after PrEP initiation vs patients without AUD

eTable 1. National Drug Codes for FDA-approved PrEP medications

eTable 2. Diagnosis codes for viral diseases treated with TDF/FTC

eTable 4. Diagnosis codes for mental health conditions

eTable 5. Diagnosis codes for substance use disorders

eTable 6. Diagnosis and service codes for STI diagnoses and testing

eTable 7. Diagnosis and service codes used to identify psychosocial therapy

eTable 8. Baseline demographic and clinical characteristics of unmatched individuals compared to matched individuals with AUD

eTable 9. Baseline demographic and clinical characteristics of unmatched cohort of PrEP users by AUD diagnosis, 2014-2021

eTable 10. Baseline demographic and clinical characteristics of PrEP users diagnosed with AUD after PrEP initiation vs. those without AUD, 2014-2021 (including unmatched and propensity score-matched samples)

## eFigure 1. CONSORT diagram showing the cohort selection of PrEP users with and without AUD diagnoses

**Individuals with claims for PrEP medications, 2014-2021**

N = 132,737

**Eligible individuals who use PrEP**

N = 43,913

**Individuals excluded**

N= 88,824

- Without 12 months continuous enrollment (6-months pre and 6-months post PrEP initiation) (N = 82,438)
- With HIV diagnosis (N = 5,960)
- With HBV diagnosis (N = 136)
- Aged <16 or >64 years (N = 290)

**Without AUD group**

N = 37,639

- TDF/FTC = 32,504 (86.36%)
- TAF/FTC = 5,135 (13.64%)
- Cabotegravir = 0 (0%)

**Pre-initiation AUD group**

N = 1,245

- TDF/FTC = 1,092 (87.71%)
- TAF/FTC = 153 (12.29%)
- Cabotegravir = 0 (0%)

**Post-initiation AUD group**

N = 5,029

- TDF/FTC = 4,454 (88.57%)
- TAF/FTC = 575 (11.43%)
- Cabotegravir = 0 (0%)

AUD, alcohol use disorder; PrEP; preexposure prophylaxis; TDF/FTC, tenofovir disoproxil fumarate/emtricitabine; TAF/FTC, tenofovir alafenamide/emtricitabine.

## eFigure 2. Love plot showing standardized mean differences before and after propensity score matching for patients with AUD diagnosis before PrEP initiation vs patients without AUD

Abbreviations: AUD, alcohol use disorder; hplan, healthcare plan; PrEP, preexposure prophylaxis; PTSD, posttraumatic stress disorder; SMD, standardized mean difference; STI, sexually transmitted infection; SUD, substance use disorder.

## eFigure 3. Love plot showing standardized mean differences before and after propensity score matching for patients with AUD diagnosis after PrEP initiation vs patients without AUD

Abbreviations: AUD, alcohol use disorder; hplan, healthcare plan; PrEP, preexposure prophylaxis; PTSD, posttraumatic stress disorder; SMD, standardized mean difference; STI, sexually transmitted infection; SUD, substance use disorder.

## eTable 1. National Drug Codes for FDA-approved PrEP medications

| **Drug** | **NDC** |
| --- | --- |
| TDF/FTC (Truvada) | 00093760756 00093770456 00378193093 00904717207 16714053401 31722056030  33342010607 35356007003 35356007006 35356007030 42291043930 42385095330 42543071904 50090087000 50090087002 50090087003 50436070101 51407011230 52959096903 54569558800 54569558802 54569558803 54868514100 55045348103 60505420203 61919066902 61958070101 65862035430 66336003203 68071211203 68180028706 68258198303 69097020902 69097074102 69238209503 70710136703 72189015602 72189022702 72189031203 72189040303 76282067730 |
| TAF/FTC (Descovy) | 61958200201 61958200202 |
| Cabotegravir (Apretude) | 49702026423 |

Abbreviations: FDA, Food and Drug Administration; NDC, National Drug Code; PrEP; preexposure prophylaxis; TDF/FTC, tenofovir disoproxil fumarate/emtricitabine; TAF/FTC, tenofovir alafenamide/emtricitabine.

## eTable 2. Diagnosis codes for viral diseases treated with TDF/FTC

|  | **ICD-9-CM codes** | **ICD-10-CM codes** |
| --- | --- | --- |
| HIV | V08  079.53  042 | B20  Z21  B97.35  O98.7 |
| HBV | 702.0  702.2  703.0 | B18.1  B19.11  B16.9  B16.2 |

Abbreviations: HBV, hepatitis B virus; HIV, human immunodeficiency virus; IDC-9-CM, *International Classification of Diseases, Ninth Revision, Clinical Modification*; IDC-10-CM, *International Classification of Diseases, Tenth Revision, Clinical Modification*; TDF/FTC, tenofovir disoproxil fumarate/emtricitabine.

**eTable 3. Diagnosis codes for alcohol use disorder**

|  | **ICD-9-CM codes** | **ICD-10-CM codes** |
| --- | --- | --- |
| Alcohol use disorder | **303 Alcohol dependence syndrome**  303.0 Acute alcoholic intoxication  303.9 Other and unspecified alcohol dependence  **291 Alcohol-induced mental disorders**  291.0 Alcohol withdrawal delirium  291.1 Alcohol-induced persisting amnestic disorder  291.2 Alcohol-induced persisting dementia  291.3 Alcohol-induced psychotic disorder with hallucinations  291.4 Idiosyncratic alcohol intoxication  291.5 Alcohol-induced psychotic disorder with delusions  291.8 Other specified alcohol-induced mental disorders  291.9 Unspecified alcohol-induced mental disorders  **305.0X Alcohol abuse, unspecified** | **F10.1 Alcohol abuse**  F10.10 uncomplicated  F10.11 in remission  F10.12 Alcohol abuse with intoxication  F10.13 Alcohol abuse, with withdrawal  F10.14 with alcohol-induced mood disorder  F10.15 Alcohol abuse with alcohol-induced psychotic disorder  F10.18 Alcohol abuse with other alcohol-induced disorders  F10.19 with unspecified alcohol-induced disorder  **F10.2 Alcohol dependence**  F10.20 uncomplicated  F10.21 in remission  F10.22 Alcohol dependence with intoxication  F10.23 Alcohol dependence with withdrawal  F10.24 with alcohol-induced mood disorder  F10.25 Alcohol dependence with alcohol-induced psychotic disorder  F10.26 with alcohol-induced persisting amnestic disorder  F10.27 with alcohol-induced persisting dementia  F10.28 Alcohol dependence with other alcohol-induced disorders  F10.29 with unspecified alcohol-induced disorder  **F10.9 Alcohol use, unspecified**  F10.90 uncomplicated  F10.91 in remission  F10.92 Alcohol use, unspecified with intoxication  F10.93 Alcohol use, unspecified with withdrawal  F10.94 with alcohol-induced mood disorder  F10.95 Alcohol use, unspecified with alcohol-induced psychotic disorder  F10.96 with alcohol-induced persisting amnestic disorder  F10.97 with alcohol-induced persisting dementia  F10.98 Alcohol use, unspecified with other alcohol-induced disorders  F10.99 with unspecified alcohol-induced disorder |

IDC-9-CM, *International Classification of Diseases, Ninth Revision, Clinical Modification*; IDC-10-CM, *International Classification of Diseases, Tenth Revision, Clinical Modification*.

## eTable 4. Diagnosis codes for mental health conditions

|  | **ICD-9-CM codes** | **ICD-10-CM codes** |
| --- | --- | --- |
| Depression | 296.2 Major depressive disorder single episode  296.3 Major depressive disorder recurrent episode  300.4 Dysthymic disorder  **311.X Depressive disorder, not elsewhere classified** | **F32.X Depressive episode**  **F33.X Major depressive disorder, recurrent**  F34.1 Dysthymic disorder |
| Anxiety | **300.X Anxiety, dissociative and somatoform disorders** | **F40.X Phobic anxiety disorders**  **F41.X Other anxiety disorders** |
| Post-traumatic stress disorder (PTSD) | 309.81 Acute PTSD | **F43.1X PTSD** |
| Bipolar disorder | 296.0 Bipolar I disorder, single manic episode  296.1 Manic disorder recurrent episode  296.4 Bipolar I disorder, most recent episode (or current) manic  296.5 Bipolar I disorder, most recent episode (or current) depressed  296.6 Bipolar I disorder, most recent episode (or current) mixed  296.7 Bipolar I disorder, most recent episode (or current) unspecified  296.8 Other and unspecified bipolar disorders | **F30.X Manic episode**  **F31.X Bipolar disorder**  F34.0 Cyclothymic disorder |

Abbreviation: IDC-9-CM, *International Classification of Diseases, Ninth Revision, Clinical Modification*; IDC-10-CM, *International Classification of Diseases, Tenth Revision, Clinical Modification*.

## eTable 5. Diagnosis codes for substance use disorders

|  | **ICD-9-CM codes** | **ICD-10-CM codes** |
| --- | --- | --- |
| Opioid use order | 305.5 Nondependent opioid abuse  304.0 Opioid type dependence | F11 Opioid related disorders |
| Nicotine dependence | 305.1: Tobacco use disorder | F17 Nicotine dependence |
| Cannabis related disorders | 305.2 Nondependent cannabis abuse  304.3 Cannabis dependence | F12 Cannabis related disorders |
| Hallucinogen related disorders | 305.3 Nondependent hallucinogen abuse  304.5 Hallucinogen dependence | F16 Hallucinogen related disorders |
| Cocaine related disorders | 304.2 Cocaine dependence  305.6 Nondependent cocaine abuse | F14 Cocaine related disorders |
| Sedative, hypnotic, or anxiolytic related disorders | 305.4 Nondependent sedative, hypnotic or anxiolytic abuse  304.1 Sedative, hypnotic or anxiolytic dependence | F13 Sedative, hypnotic, or anxiolytic related disorders |
| Stimulant related disorders | 969.7 Poisoning by psychostimulants | F15 Other stimulant related disorders |
| Other drug use disorders | 305.8 Nondependent antidepressant type abuse  305.9 Nondependent other mixed or unspecified drug abuse  304.6 Other specified drug dependence  304.7 Combinations of opioid type drug with any other drug dependence  304.8 Combinations of drug dependence excluding opioid type drug  304.9 Unspecified drug dependence  292: Drug-induced mental disorders | F18 Inhalant related disorders  F19 Other psychoactive substance related disorders |
| Opioid overdose | 965.00 Poisoning by opium (alkaloids), unspecified  965.02 Poisoning by methadone  965.09 Poisoning by other opiates and related narcotics  E850.1 Accidental poisoning by methadone  E850.2 Accidental poisoning by other opiates and related narcotics  E850.0 Accidental poisoning by heroin  E935.0 Heroin causing adverse effects in therapeutic use  E935.1 Methadone causing adverse effects in therapeutic use  E935.2 Other opiates and related narcotics causing adverse effects in therapeutic use | T40.0 Poisoning by, adverse effect of and underdosing of opium  T40.1 Poisoning by and adverse effect of heroin  T40.2 Poisoning by, adverse effect of and underdosing of other opioids  T40.3 Poisoning by, adverse effect of and underdosing of methadone  T40.4 Poisoning by, adverse effect of and underdosing of other synthetic narcotics  T40.6 Poisoning by, adverse effect of and underdosing of other and unspecified narcotics |
| Amphetamine overdose | 305.7 Nondependent amphetamine or related acting sympathomimetic abuse  304.4 Amphetamine and other psychostimulant dependence | T43.62 Poisoning by, adverse effect of and underdosing of amphetamines |
| Cocaine overdose | 970.8 Poisoning by other specified central nervous system stimulants | T40.5 Poisoning by, adverse effect of and underdosing of cocaine |

Abbreviations: IDC-9-CM, *International Classification of Diseases, Ninth Revision, Clinical Modification*; IDC-10-CM, *International Classification of Diseases, Tenth Revision, Clinical Modification*.

## eTable 6. Diagnosis and service codes for STI diagnoses and testing

|  | **ICD-9-CM codes** | **ICD-10-CM codes** | **CPT** |
| --- | --- | --- | --- |
| STI | 054.1 Genital herpes  091-099 Other STIs  070 Viral hepatitis  079.4 HPV  131 Trichomoniasis  614.0 Acute salpingitis and oophoritis  614.3 Acute parametritis and pelvic cellulitis  614.5 Acute or unspecified pelvic peritonitis, female  615.0 Acute inflammatory diseases of uterus, except cervix  795.05 Cervical HPV DNA test positive  795.09 Other abnormal Papanicolaou smear of cervix and cervical HPV  795.15 Vaginal HPV DNA test positive  795.19 Other abnormal Papanicolaou smear of vagina and vaginal HPV  796.75 Anal HPV DNA test positive  V01.6 Contact with or exposure to venereal diseases | A51-A64 Other STIs  B15-B19 Viral hepatitis  N73 Pelvic inflammatory disease  R85.81-R85.82 Anal HPV DNA test positive  R87.81-R87.82 HPV DNA test positive from female genital organs  Z20.2 Contact with and (suspected) exposure to infections with a predominantly sexual mode of transmission  Z20.5 Contact with and (suspected) exposure to viral hepatitis |  |
| STI testing or screening | V65.45 Counseling on other sexually transmitted diseases  V73.8 Screening examination for other specified viral and chlamydial diseases  V73.9 Screening examination for unspecified viral and chlamydial disease  V74.5 Screening examination for venereal disease | Z11.3 encounter for screening for infections with a predominantly sexual mode of transmission  Z11.5 Encounter for screening for other viral diseases  Z11.8 encounter for screening for other infectious and parasitic diseases | 86781, 86780, 86592, 86593, 87164 Syphilis  86631, 86632, 87110, 87270, 87320, 87490-87492, 87590–87592, 87810, 87850 Chlamydia and gonorrhea  87273, 87274, 87528, 87529, 87530, 86696, 87207 HSV  86729 LGV  87620, 87621, 87622, 88175 HPV  80074, 86704–86707, 87340-87341, 87350, 87515, 87516, 87517 HBV  80074, 86803, 86804, 87520–87522 HCV  87070, 87808, 87810, 87850, 87660 Trichomoniasis |

Abbreviations: CPT, Common Procedural Terminology; HBV, hepatitis B virus; HCV; hepatitis C virus; HPV, human papillomavirus; HSV, herpes simplex virus; IDC-9-CM, *International Classification of Diseases, Ninth Revision, Clinical Modification*; IDC-10-CM, *International Classification of Diseases, Tenth Revision, Clinical Modification*; LGV, lymphogranuloma venereum; STI, sexually transmitted infection.

## eTable 7. Diagnosis and service codes used to identify psychosocial therapy

|  | **ICD-procedure, CPT, and HCPCS codes** | **MarketScan codes** |
| --- | --- | --- |
| Psychotherapy | 90832-90838, 90845, 90847-90849, 90853, 90857, 90865, 90875, 90876, 90880, 90900-90902, 90904, 90906, 90908, 90910, 99510, H0004, H0005, H2019, H2020, H2032, H5010, H5020, H5025, T1006, T1012 | PROCGRP=135, 136, 137 |
| Alcoholism counseling | V65.42, Z71.41, G0396, G0397, G0443, H0001, H0050, H0015, H0021, H0030 |  |

Abbreviations: CPT, Common Procedural Terminology; HCPCS, Healthcare Common Procedure Coding System; IDC-9-CM, *International Classification of Diseases, Ninth Revision, Clinical Modification*; IDC-10-CM, *International Classification of Diseases, Tenth Revision, Clinical Modification*; PROCGRP, procedure group.

## eTable 8. Baseline demographic and clinical characteristics of unmatched individuals compared to matched individuals with AUD

|  | **Unmatched exposed group (N=92)** | **Matched exposed group**  **(N=1153)** | **Total exposed group**  **(N=1245)** | **P value** |
| --- | --- | --- | --- | --- |
| **Age, in years** |  |  |  |  |
| Mean (SD) | 29.6 (9.92) | 35.3 (11.45) | 34.9 (11.44) | <0.001^1^ |
| Median | 27.0 | 33.0 | 33.0 |  |
| Range | 16.0, 58.0 | 16.0, 64.0 | 16.0, 64.0 |  |
| **Sex assigned at birth**, n (%) |  |  |  | <0.001^2^ |
| Male | 30 (32.6%) | 178 (15.4%) | 208 (16.7%) |  |
| Female, (%) | 62 (67.4%) | 975 (84.6%) | 1037 (83.3%) |  |
| **Region, n (%)** |  |  |  | 0.2008^2^ |
| Northeast | 33 (35.9%) | 315 (27.5%) | 348 (28.1%) |  |
| Midwest | 15 (16.3%) | 160 (13.9%) | 175 (14.1%) |  |
| South | 24 (26.1%) | 404 (35.2%) | 428 (34.5%) |  |
| West | 20 (21.7%) | 268 (23.4%) | 288 (23.2%) |  |
| Unknown | 0 (0%) | 6 (0.005%) | 6 (0.005%) |  |
| **Insurance plan, n (%)** |  |  |  | 0.5312^2^ |
| HMO | 10 (10.9%) | 166 (14.4%) | 176 (14.1%) |  |
| POS | 14 (15.2%) | 217 (18.8%) | 231 (18.6%) |  |
| PPO | 49 (53.3%) | 535 (46.4%) | 584 (46.9%) |  |
| Others | 19 (20.7%) | 235 (20.4%) | 254 (20.4%) |  |
| **Employed, n (%)** | 72 (78.3%) | 914 (79.3%) | 986 (79.2%) | 0.8182^2^ |
| **Depression, n (%)** | 73 (79.3%) | 316 (27.4%) | 389 (31.2%) | <0.001^2^ |
| **Anxiety, n (%)** | 53 (57.6%) | 280 (24.3%) | 333 (26.7%) | <.0001^2^ |
| **Schizophrenia, n(%)** | 1 (1.1%) | 9 (0.8%) | 10 (0.8%) | 0.7514^2^ |
| **Bipolar, n (%)** | 44 (47.8%) | 118 (10.2%) | 162 (13.0%) | <0.001^2^ |
| **PTSD, n (%)** | 18 (19.6%) | 76 (6.6%) | 94 (7.6%) | <0.001^2^ |
| **Substance use disorders, n (%)** | 91 (98.9%) | 317 (27.5%) | 408 (32.8%) | <0.001^2^ |
| **STI, n (%)** | 7 (7.6%) | 125 (10.8%) | 132 (10.6%) | 0.3325^2^ |
| **STI screening n (%)** | 23 (25.0%) | 166 (14.4%) | 189 (15.2%) | 0.0064^2^ |
| **Psychotherapy n (%)** | 23 (25.0%) | 150 (13.0%) | 173 (13.9%) | 0.0014^2^ |

^1^Kruskal-Wallis

^2^Chi-Square

AUD, alcohol use disorder; HMO, health maintenance organization; PrEP, preexposure prophylaxis; POS, point of service; PPO, preferred provider organization; STI, sexually transmitted infection.

## eTable 9. Baseline demographic and clinical characteristics of unmatched cohort of PrEP users by AUD diagnosis, 2014-2021

| **Characteristic** | **PrEP users with AUD diagnosis** | | **PrEP users without AUD diagnoses**  (N = 37,639) | **P value**^a^ | |
| --- | --- | --- | --- | --- | --- |
|  |  |  |  | ***Pre-initiation group*** | ***Post-initiation group*** |
|  | ***Pre-initiation group***  (N = 1,245) | ***Post-initiation group***  (N = 5,029) |  |  |  |
| **Age in years, mean (SD)** | 34.88 (11.43) | 35.49 (11.96) | 35.85 (10.78) | <0.01 | 0.03 |
| **Male, n (%)** | 1,037 (83.29) | 4,345 (86.40) | 33,990 (90.31) | <0.01 | <0.01 |
| **Insurance plan, n (%)** |  |  |  | 0.32 | <0.01 |
| HMO | 176 (14.14) | 773 (15.37) | 5,015 (13.32) |  |  |
| POS | 231 (18.55) | 700 (13.92) | 7,539 (20.03) |  |  |
| PPO | 584 (46,91) | 2,407 (47.86) | 16,987 (45.13) |  |  |
| Others | 254 (20.4) | 1,149 (22.85) | 8,098 (21.51) |  |  |
| **Employed, n (%)** | 986 (79.20) | 4,109 (81.71) | 31,409 (83.45) | <0.01 | <0.01 |
| **Region, n (%)** |  |  |  | 0.07 | <0.01 |
| Northeast | 348 (27.95) | 1,342 (26.69) | 9,531 (25.32) |  |  |
| Midwest | 175 (14.06) | 806 (16.03) | 4,833 (12.84) |  |  |
| South | 428 (34.38) | 1,758 (34.96) | 13,897 (36.92) |  |  |
| West | 288 (23.13) | 1,112 (22.11) | 9,253 (24.58) |  |  |
| Unknown | 6 (0.48) | 11 (0.22) | 125 (0.33) |  |  |
| **Depression, n (%)** | 389 (31.24) | 1,357 (26.98) | 1996 (5.30) | <0.01 | <0.01 |
| **Anxiety, n (%)** | 333 (26.75) | 1,186 (23.58) | 2,488 (6.61) | <0.01 | <0.01 |
| **Bipolar, n (%)** | 162 (13.01) | 547 (10.88) | 590 (1.57) | <0.01 | <0.01 |
| **Substance use disorders** |  |  |  |  |  |
| Opioids**,** n (%) | 65 (5.22) | 259 (5.15) | 90 (0.24) | <0.01 | <0.01 |
| Nicotine**,** n (%) | 164 (13.17) | 497 (9.88) | 476 (1.26) | <0.01 | <0.01 |
| Cannabis**,** n (%) | 121 (9.72) | 538 (10.70) | 116 (0.31) | <0.01 | <0.01 |
| Stimulants**,** n (%) | 100 (8.03) | 298 (5.93) | 103 (0.27) | <0.01 | <0.01 |
| Sedatives**,** n (%) | 64 (5.14) | 204 (4.06) | 29 (0.08) | <0.01 | <0.01 |
| Other**,** n (%) | 70 (5.62) | 280 (5.57) | 99 (0.26) | <0.01 | <0.01 |
| **STI n (%)** | 73 (5.86) | 174 (3.46) | 2,757 (7.32) | 0.05 | <0.01 |
| **STI screening n (%)** | 60 (4.82) | 119 (2.37) | 4,188 (11.13) | <0.01 | <0.01 |
| **Psychotherapy n (%)** | 173 (13.90) | 535 (10.64) | 2,084 (5.54) | <0.01 | <0.01 |

Comparisons were made between PrEP users with AUD diagnoses before or after initiation and PrEP users without AUD diagnoses. Statistical differences between the two groups were evaluated using Student’s t-test for continuous variables, Pearson’s chi-square test for binary variables, and Mantel-Haenszel chi-square test for nominal variables.

AUD, alcohol use disorder; HMO, health maintenance organization; PrEP, preexposure prophylaxis; POS, point of service; PPO, preferred provider organization; STI, sexually transmitted infection.

## eTable 10. Baseline demographic and clinical characteristics of PrEP users diagnosed with AUD after PrEP initiation vs. those without AUD, 2014-2021 (including unmatched and propensity score-matched samples)

| **Characteristics** | **UNMATCHED** |  |  | **MATCHED** |  |  |
| --- | --- | --- | --- | --- | --- | --- |
|  | ***Post-initiation group***  (N=5,029) | ***Without AUD group***  (N = 37,639) | ***SMD***^a^ | ***Post-initiation group***  (N = 4,303) | ***Without AUD group***  (N=4,303) | ***SMD***^a^ |
| **Age in years, mean (SD)** | 35.5 (11.9) | 35.9 (10.7) | 0.031 | 36.0 (11.9) | 36.5 (11.5) | 0.042 |
| **Male, n (%)** | 4,345 (86.4) | 33,990 (90.3) | 0.122 | 3762 (87.4) | 3794 (88.2) | 0.023 |
| **Insurance plan, n (%)** |  |  | 0.035 |  |  | 0.008 |
| HMO | 773 (15.3) | 5,015 (13.3) |  | 656 (15.2) | 613 (14.2%) |  |
| POS | 700 (13.9) | 7,539 (20.0) |  | 610 (14.2) | 688 (16.0%) |  |
| PPO | 2,407 (47.8) | 16,987 (45.1) |  | 2066 (48.0) | 2073 (48.2%) |  |
| Others | 1,149 (22.8) | 8,098 (21.5) |  | 971 (22.6) | 929 (21.6%) |  |
| **Employed, n (%)** | 4,109 (81.7) | 31,409 (83.4) | 0.046 | 3525 (81.9) | 3507 (81.5%) | 0.011 |
| **Region, n (%)** |  |  | N/A |  |  | N/A |
| Northeast | 1,342 (26.7) | 9,531 (25.4) |  | 1161 (27.0) | 1173 (27.3%) |  |
| Midwest | 806 (16.1) | 4,833 (12.9) |  | 661 (15.4) | 610 (14.2%) |  |
| South | 1,758 (35.0) | 13,897 (37.0) |  | 1515 (35.3) | 1514 (35.3%) |  |
| West | 1,112 (22.2) | 9,253 (24.7) |  | 959 (22.3) | 994 (23.2%) |  |
| Unknown | 11 | 125 |  | 7 | 12 |  |
| **Depression, n (%)** | 1,357 (27.0) | 1996 (5.3) | 0.617 | 917 (21.3) | 997 (23.2%) | 0.053 |
| **Anxiety, n (%)** | 1,186 (23.6) | 2,488 (6.6) | 0.488 | 866 (20.1) | 900 (20.9%) | 0.008 |
| **PTSD, n (%)** | 317 (6.3) | 738 (2.0) |  | 194 (4.5) | 170 (4.0%) |  |
| **Bipolar, n (%)** | 547 (10.9) | 590 (1.6) | 0.393 | 305 (7.1) | 328 (7.6%) | 0.022 |
| **Schizophrenia, n (%)** | 39 (0.8) | 28 (0.1) | 0.108 | 18 (0.4%) | 12 (0.3%) | 0.021 |
| **Substance use disorders** |  |  | 0.785 |  |  | 0.026 |
| Opioids**,** n (%) | 259 (5.1) | 90 (0.24) |  | 170 (4.0) | 46 (1.1) |  |
| Nicotine**,** n (%) | 497 (9.8) | 476 (1.3) |  | 255 (5.9) | 434 (10.1) |  |
| Cannabis**,** n (%) | 538 (10.7) | 116 (0.3) |  | 255 (5.9) | 107 (2.5) |  |
| Stimulants**,** n (%) | 298 (5.9) | 103 (0.2) |  | 152 (3.5) | 91 (2.1) |  |
| Sedatives**,** n (%) | 204 (4.0) | 29 (0.1) |  | 98 (2.3) | 27 (0.6) |  |
| Other**,** n (%) | 280 (5.5) | 99 (0.3) |  | 128 (3.0) | 91 (2.1) |  |
| **STI, n (%)** | 297 (5.9) | 6,700 (17.8) | 0.374 | 275 (6.4) | 282 (6.6) | 0.005 |
| **STI screening, n (%)** | 579 (11.5) | 3,806 (10.1) | 0.045 | 475 (11.0) | 505 (11.7) | 0.022 |
| **Psychotherapy, n (%)** | 535 (10.6) | 2,084 (5.5) | 0.188 | 431 (10.0) | 484 (11.2) | 0.045 |

^a^An SMD <0.1 shows adequate covariate balance.

^b^Region was excluded from the propensity score model because of insufficient sample size in the unknown group.

Abbreviations: AUD, alcohol use disorder; HMO, health maintenance organization; PrEP, preexposure prophylaxis; POS, point of service; PPO, preferred provider organization; PTSD, posttraumatic stress disorder; SMD, standardized mean difference; STI, sexually transmitted infection.
